# Long-term prognosis after stapled and hand-sewn ileal pouch–anal anastomoses for familial adenomatous polyposis: a multicenter retrospective study

**DOI:** 10.1007/s00384-024-04608-9

**Published:** 2024-03-02

**Authors:** Kyota Tatsuta, Mayu Sakata, Moriya Iwaizumi, Kazuya Okamoto, Shigeto Yoshii, Masashi Mori, Yutaro Asaba, Takashi Harada, Mikihiro Shimizu, Kiyotaka Kurachi, Hiroya Takeuchi

**Affiliations:** 1 Department of Surgery, Hamamatsu University School of Medicine, 1-20-1, Handayama, Higashi-ku, Hamamatsu, Shizuoka 431-3192 Japan; 2 Department of Laboratory Medicine, Hamamatsu University School of Medicine, 1-20-1 Handayama, Higashi-ku, Hamamatsu Shizuoka, 431-3192 Japan; 3 Department of Surgery, Fujieda Municipal General Hospital, 4-1-11 Surugadai, Fujieda, Shizuoka 426-8677 Japan; 4 Department of Gastroenterology, Fujieda Municipal General Hospital, 4-1-11 Surugadai, Fujieda, Shizuoka 426-8677 Japan; 5 Department of Gastroenterology, Fujinomiya City General Hospital, 3-1 Nishiki-cho, Fujinomiya Shizuoka, 418-0076 Japan; 6 Department of Surgery, JA Shizuoka Kohseiren Enshu Hospital, 1-1-1 Chuou, Naka-ku, Hamamatsu, 430-0929 Japan; 7 Department of Surgery, Hamamatsu Medical Center, 328, Tomitsuka, Naka-ku, Hamamatsu, Tomitsuka 432-8580 Japan; 8 Center for Clinical Research, Hamamatsu University Hospital, 1-20-1 Handayama, Higashi-ku, Hamamatsu, Shizuoka 431-3192 Japan

**Keywords:** Familial adenomatous polyposis, Hand-sewn ileal pouch–anal anastomosis, Stapled ileal pouch–anal anastomosis, Metachronous remnant rectal cancer, Long-term prognosis

## Abstract

**Purpose:**

The long-term prognosis of stapled and hand-sewn ileal pouch–anal anastomoses in familial adenomatous polyposis patients in Japan remains unknown. This study aimed to compare the overall survival in familial adenomatous polyposis patients who underwent stapled or hand-sewn ileal pouch–anal anastomosis.

**Methods:**

This multicenter retrospective study was conducted at 12 institutions in Shizuoka Prefecture, Japan. The clinical outcomes of 53 eligible familial adenomatous polyposis patients who underwent stapled (*n* = 24) and hand-sewn (*n* = 29) ileal pouch–anal anastomosis were compared.

**Results:**

The median follow-up duration was 171.5 months. The incidence of adenoma in the remnant rectum or anal transitional zone and metachronous rectal cancer was significantly more common in stapled ileal pouch–anal anastomosis (adenoma: stapled, 45.8%, vs. hand-sewn, 10.3%, *p* = 0.005; metachronous rectal cancer: 29.2%, vs. none, *p* = 0.002). The number of deaths was remarkably higher in stapled ileal pouch–anal anastomosis (*p* = 0.002). Metachronous rectal cancer was the most common cause of death. Overall survival was worse in stapled ileal pouch–anal anastomosis than in hand-sewn ileal pouch–anal anastomosis (120 months, 90.7% vs. 96.6%; 240 months, 63.7% vs. 96.6%; *p* = 0.044). Cox regression analysis revealed the independent effects of preoperative advanced colorectal cancer and stapled ileal pouch–anal anastomosis on overall survival.

**Conclusion:**

Stapled ileal pouch–anal anastomosis negatively affected the overall survival of familial adenomatous polyposis patients. Therefore, hand-sewn ileal pouch–anal anastomosis is recommended for better prognosis in these patients.

**Supplementary Information:**

The online version contains supplementary material available at 10.1007/s00384-024-04608-9.

## Introduction

Familial adenomatous polyposis (FAP) is a genetic disease that typically develops multiple colon polyps until the patients reach their 20s and eventually harbor colorectal cancer at 100% penetrance until their 60s [1, 2]. Chemoprevention or intensive endoscopic resection may have antineoplastic effects on the recurrence of colonic adenomas [3–5]; however, prophylactic surgery of the colon and rectum is the only curative treatment for FAP.

The choice of surgical procedure is determined by various factors, including the genotype of adenomatous polyposis coli (APC), number of polyps, age, the presence of cancer, fertility, and quality of life [6, 7]. From the perspective of long-term prognosis, colectomy with ileorectal anastomosis is associated with a higher risk of metachronous remnant rectal cancer and a poorer prognosis than total proctocolectomy with ileal pouch–anal anastomosis (IPAA) because of the remnant rectum [8]. At present, IPAA is considered the standard surgical procedure for minimizing the risk of rectal cancer death and is recommended in the Japanese guidelines for treating FAP patients [9–11].

IPAA is divided into the hand-sewn technique, with mucosectomy down to the dentate line, and the stapled technique, without mucosectomy. Recent retrospective studies have reported differences in the occurrence of remnant rectal and anal transitional zone (ATZ) adenomas between stapled and hand-sewn IPAA [12, 13]. Rectal adenomas were found significantly more often after stapled than after hand-sewn anastomosis [12, 13]. Colorectal carcinogenesis in FAP follows a conventional adenoma-carcinoma sequence; thus, the long-term prognosis in IPAA may depend on whether stapled or hand-sewn anastomosis is used. However, owing to the short surveillance duration in previous studies, carcinogenesis in the remnant rectum and the long-term prognosis of IPAA using different anastomosis methods is unknown [14, 15].

We hypothesized that hand-sewn IPAA contributes to good long-term outcomes. The present study aimed to compare the long-term prognosis between FAP patients who underwent stapled IPAA and those who had hand-sewn IPAA.

## Materials and methods

### Study design and patient population

The original data for this study were compiled from 12 institutions accredited by the Japan Surgical Society or the Japanese Society of Gastroenterology in Shizuoka Prefecture. The data of all patients diagnosed with FAP who underwent colorectal resection at each institution from 1987 to 2023 were retrospectively collected and registered in the database. FAP was defined according to the following three criteria: (i) 100 or more adenomatous polyps in the colon with or without a family history of FAP, (ii) fewer than 100 adenomatous polyps in the colon with a family history of FAP, and (iii) germline mutations in the *APC* gene. The diagnostic criteria adhered to Japanese Society for Cancer of the Colon and Rectum guidelines for the Clinical Practice of Hereditary Colorectal Cancer [16, 17]. The characteristics of variants in genetically diagnosed cases were showed according to the gene-specific ACMG/AMP classification criteria for germline APC variants [18] (Supplementary Table 1). We defined FAP patients with ≥ 1001, 100–1000, and ≤ 99 polyps in the colon and rectum as having profuse, sparse, and attenuated phenotypes, respectively. This retrospective observational cohort study was approved by the institutional review board of our university. Opt-out or written consent was obtained from the patients. The data of all patients who underwent IPAA were extracted from the database. We analyzed patients from the collected cases that could be followed up, including those who temporarily dropped out of surveillance.

### Surgical procedure

**Fig. 1:**
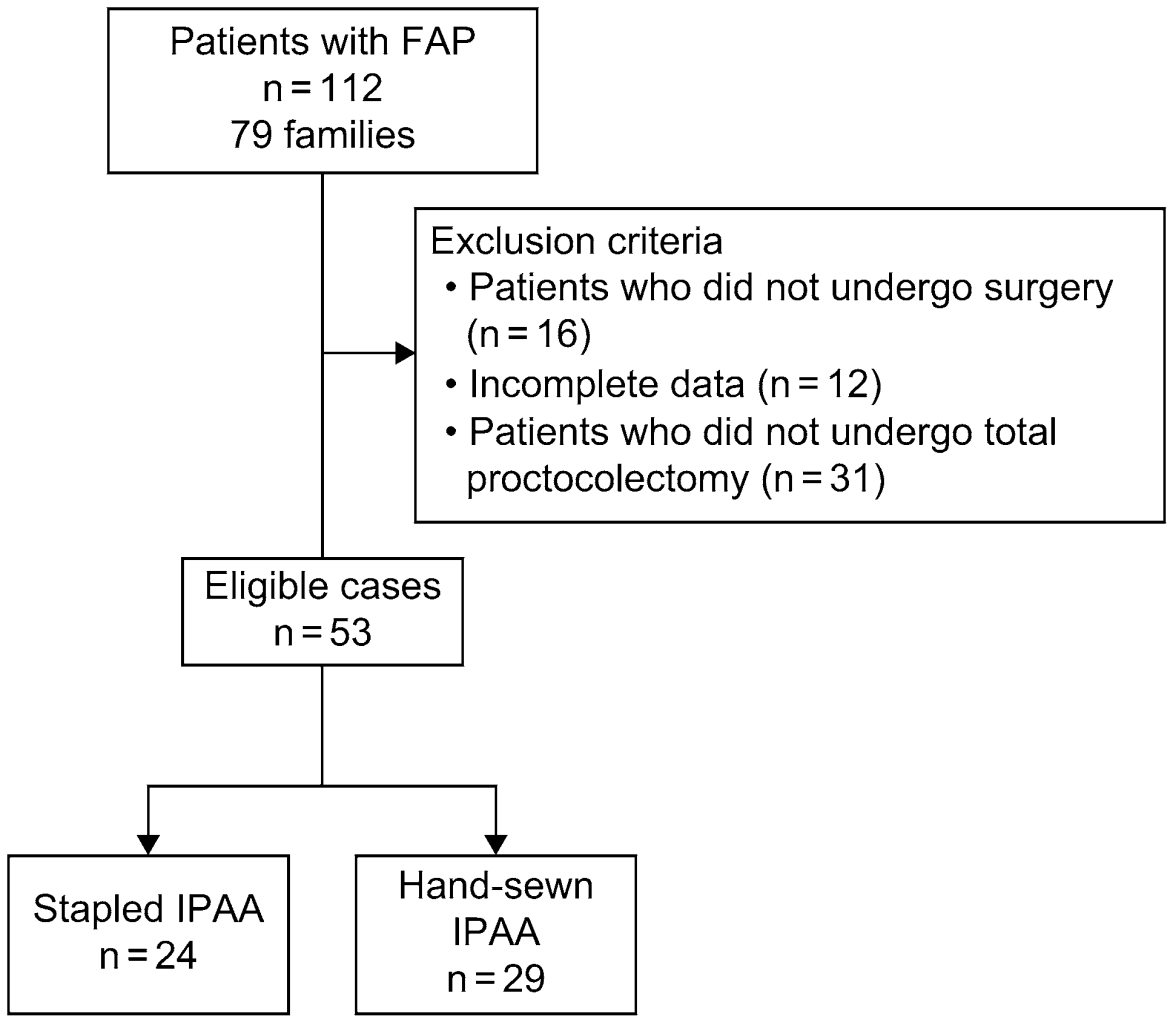
Flow diagram of the present study. FAP, familial adenomatous polyposis

Surgery was performed for either prophylactic reasons or due to cancer. The staging of cancer was classified into early-stage (T1N0M0 or T1N + M0) and advanced (T2-4N0M0 or T2-4N + M0) cancers, in accordance with the Union for International Cancer Control TNM classification of malignant tumors, 8th edition [19], and Japanese Classification of Colorectal, Appendiceal, and Anal Carcinoma, 3rd English Edition [20]. All surgeries were performed by or under the supervision of surgeons with sufficient experience in FAP. Hand-sewn IPAA was the standard procedure. Rectal preservation was selected under the following conditions: fewer than 20 rectal adenomas, rectal adenomas of ≤ 1 cm, absence of high-grade dysplasia or cancer, and young women without definitive offspring. There were no established selection criteria for choosing between stapled and hand-sewn IPAA based on cases involving rectal polyps or malignancy. Stapled IPAA was performed in patients for whom hand-sewn IPAA was preferable but who were unable to accept defecation disorder. Additionally, patients in whom preoperative assessment suggested that hand-sewn IPAA would be challenging due to the body size or other factors opted for stapled IPAA. The final decision on the surgical procedure was left to the discretion of each institution. The patients were divided into two groups (stapled IPAA, *n* = 24; hand-sewn IPAA, *n* = 29; Fig. 1).

### Postoperative surveillance

Patients were recommended to undergo imaging examinations with esophagogastroduodenoscopy and colonoscopy once a year and computed tomography every few years; however, the number varied according to era and surgical procedure. Post-operative surveillance was conducted at each institution. Pouchoscopy was used to determine whether the rectum mucosa remained and to measure the length of the remnant rectum. Overall survival (OS) was calculated from the time of surgery to the day of death. Patients were followed-up until death or until the end of the study (December 31, 2023). Patients who interrupted follow-up were recognized as censored, and their OS was calculated based on the number of days until censoring. The cumulative risk of adenoma and metachronous remnant rectal cancer in the remnant rectum or ATZ was similarly calculated. We conducted a sensitivity analysis for all eligible patients (stapled IPAA, *n* = 20; hand-sewn IPAA, *n* = 20) after excluding those with interrupted follow-up.

### Statistical analyses

Statistical analyses were performed using JMP® 16 (SAS Institute Inc., Cary, NC, USA). Continuous variables are presented as medians and ranges and tested using the Mann–Whitney *U* test. Categorical data are expressed as numbers and frequencies and analyzed using Fisher’s exact test. The cumulative risks of adenoma and metachronous remnant rectal cancer in the remnant rectum or ATZ and OS were calculated using the Kaplan–Meier method and log-rank test. For long-term prognosis, we performed a Gray test accounting for deaths unrelated to FAP-related malignancies or surgery as a competing risk. Univariable and multivariable comparisons of survival times were performed using Cox regression analysis. Factors with a *p* value < 0.05 on univariable analysis were included in a multivariable analysis. Data with *p* values < 0.05 were regarded as statistically significant. The post hoc analysis showed a power of 81% for OS of eligible cases using Cox proportional hazards model with a two-sided significance level of *p* < 0.05.

## Results

### Clinical characteristics

The participants’ characteristics are presented in Table 1. The median age of all eligible patients at surgery was 33 years. Most patients had a profuse or sparse phenotype, and prevention was the most common surgical indication in both groups. There were no significant differences between the clinical characteristics of the two groups.

**Table 1:** Clinical characteristics of the study participants

	**Stapled** ***n* = 24**	**Hand-sewn** ***n* = 29**	***P* value**
Age at surgery, years, median (range)	34.5 (16–59)	32 (16–56)	0.128
Sex			0.237
Male, cases (%)	11 (45.8)	18 (62.1)	
Female, cases (%)	13 (54.2)	11 (37.9)	
Family history			0.170
Yes, cases (%)	12 (50)	16 (55.2)	
No, cases (%)	3 (12.5)	8 (27.6)	
Unknown, cases (%)	9 (37.5)	5 (17.2)	
Phenotype, cases (%)			0.241
Profuse (polyps ≥ 1001)	15 (62.5)	12 (41.4)	0.241
Sparse (polyps 100–1000)	9 (37.5)	16 (55.2)	
Attenuated (polyps ≤ 99)	0 (0)	1 (3.4)	
Indication for surgery, cases (%)			
Prevention	14 (58.3)	18 (62.1)	1.000
Cancer			
Early colorectal cancer	5 (20.8)	9 (31)	0.535
T1N0M0	4 (16.7)	8 (27.6)	0.512
T1N + M0	1 (4.2)	1 (3.4)	1.000
Advanced colorectal cancer	5 (20.8)	6 (20.7)	1.000
T2-4N0M0	2 (8.3)	2 (6.9)	1.000
T2-4N + M0	3 (12.5)	4 (13.8)	1.000
The location of cancer, cases (%)			
Colon	4 (16.7)	11 (37.9)	0.127
Rectum	6 (25.0)	4 (13.8)	0.482
The length of the remnant rectum, cm, median (range)*	2.0 (1.0–3.0)	0	< 0.001

*One case of missing data in the stapled group

### Postoperative surveillance

The details of the postoperative surveillance are presented in Table 2. The median surveillance period of all eligible patients was 171.5 months. The percentage of all patients lost to follow-up was 15.1%. The incidence of adenoma in the remnant rectum or ATZ was significantly higher after stapled IPAA than after hand-sewn IPAA. The three cases in hand-sewn IPAA were adenomas caused by mucosal islets. The incidence of metachronous remnant rectal cancer was significantly higher in stapled than in hand-sewn IPAA. Other FAP-related malignancies did not differ between the two groups. The number of deaths during the surveillance period was significantly higher in stapled than in hand-sewn IPAA. Metachronous remnant rectal cancer was the most common cause of death in patients undergoing stapled IPAA.

**Table 2:** Results of postoperative surveillance

	**Stapled** ***n* = 24**	**Hand-sewn** ***n* = 29**	***P* value**
Median follow-up period after surgery, months (range)	198.7 (13–447.8)	149.0 (7.9–539.5)	0.163
Follow up status			0.017
End of follow-up*, cases (%)	1 (4.2)	4 (13.8)	
Lost to follow-up, cases (%)	3 (12.5)	5 (17.2)	
Followed up, cases (%)	11 (45.8)	19 (65.5)	
Dead, cases (%)	9 (37.5)	1 (3.4)	
Temporary drop-out from surveillance, cases (%)	2 (8.3)	2 (6.9)	1.000
Adenoma in the remnant rectum or ATZ, cases (%)	11 (45.8)	3 (10.3)	0.005
FAP-related malignancies			
Metachronous remnant rectal cancer, cases (%)	7 (29.2)	0 (0)	0.002
Gastric cancer, cases (%)	4 (16.7)	1 (4.2)	0.164
Duodenal cancer, cases (%)	2 (8.3)	2 (6.9)	1.000
Thyroid cancer, cases (%)	0 (0)	1 (4.2)	1.000
Pouch cancer, cases (%)	0 (0)	2 (6.9)	0.495
Desmoid tumor, cases (%)	6 (25)	8 (17.6)	1.000
Malignancies not related to FAP			
Lung cancer	0 (0)	2 (6.9)	0.495
Uterine cancer	1 (4.2)	0 (0)	0.453
Cause of death, cases (%)			
FAP-related malignancies, cases (%)	6 (25)	1 (3.4)	0.038
Metachronous rectal cancer, cases (%)	4 (16.7)	0 (0)	0.036
Preoperative colorectal cancer, cases (%)	1 (4.2)	1 (3.4)	1.000
Others, cases (%)	3 (12.5)	0 (0)	0.106

*ATZ* anal transitional zone, *FAP* familial adenomatous polyposis

*Patients who are over 75 years of age or wish to end surveillance

**Fig. 2:**
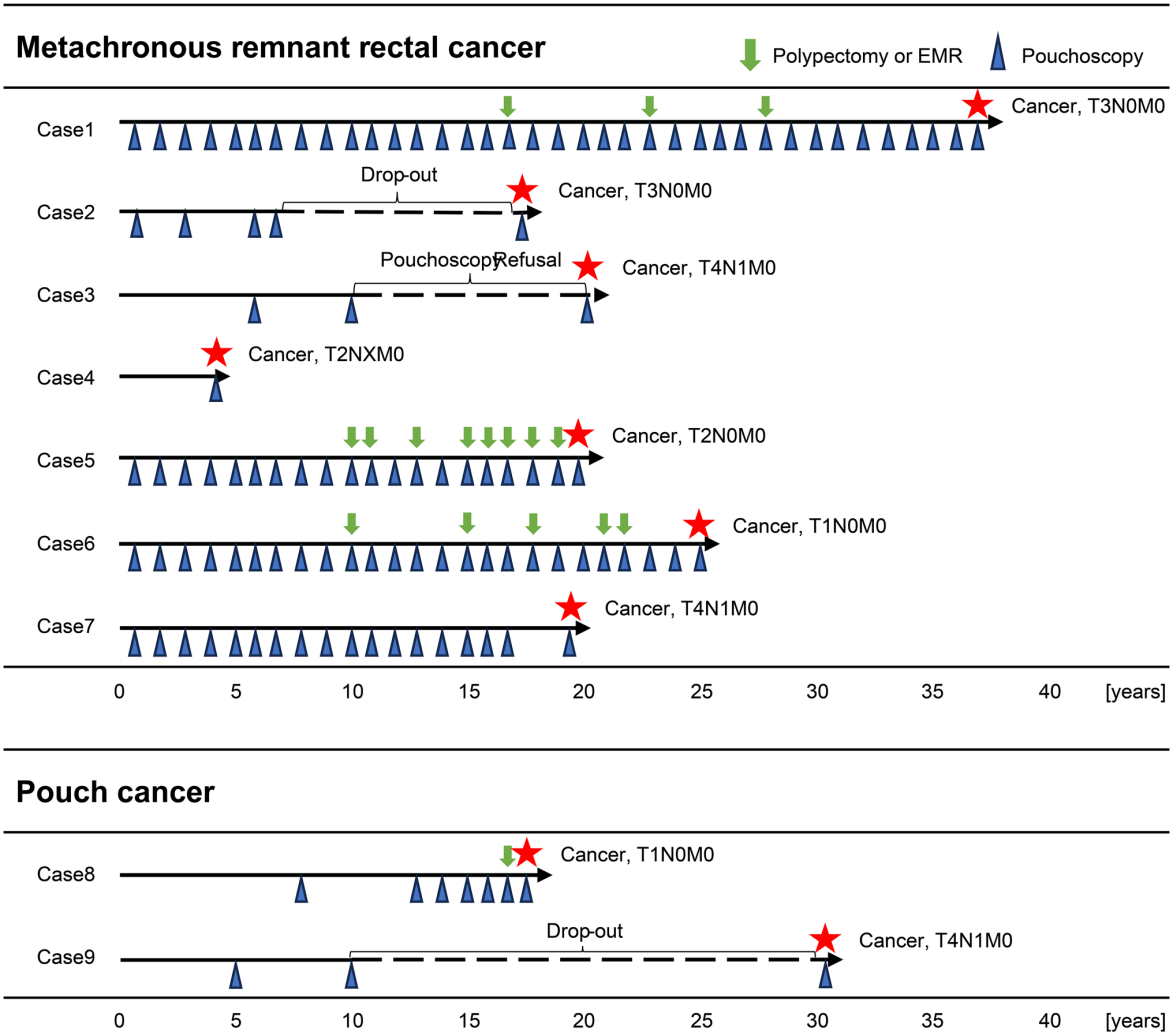
Detailed follow-up data on patients with metachronous remnant rectal or pouch cancer. The vertical axis is organized chronologically according to the diagnosis dates for metachronous remnant rectal cancer and pouch cancer. The horizontal axis represents the follow-up period, with the date of the total proctocolectomy marked as zero. EMR, endoscopic mucosal resection

Figure 2 presents detailed postoperative surveillance on nine patients who developed metachronous remnant rectal cancer or pouch cancer. Among the nine patients, three underwent annual pouchoscopy but still developed metachronous carcinogenesis. The other cases developed metachronous carcinogenesis after a period exceeding 2 years between pouchoscopies. The extended interval of over 10 years between pouchoscopies in three cases was attributed to temporary drop-out in two cases and refusal of pouchoscopy in one.

### Cumulative risk of adenoma formation and carcinogenesis in the remnant rectum or ATZ

**Fig. 3:**
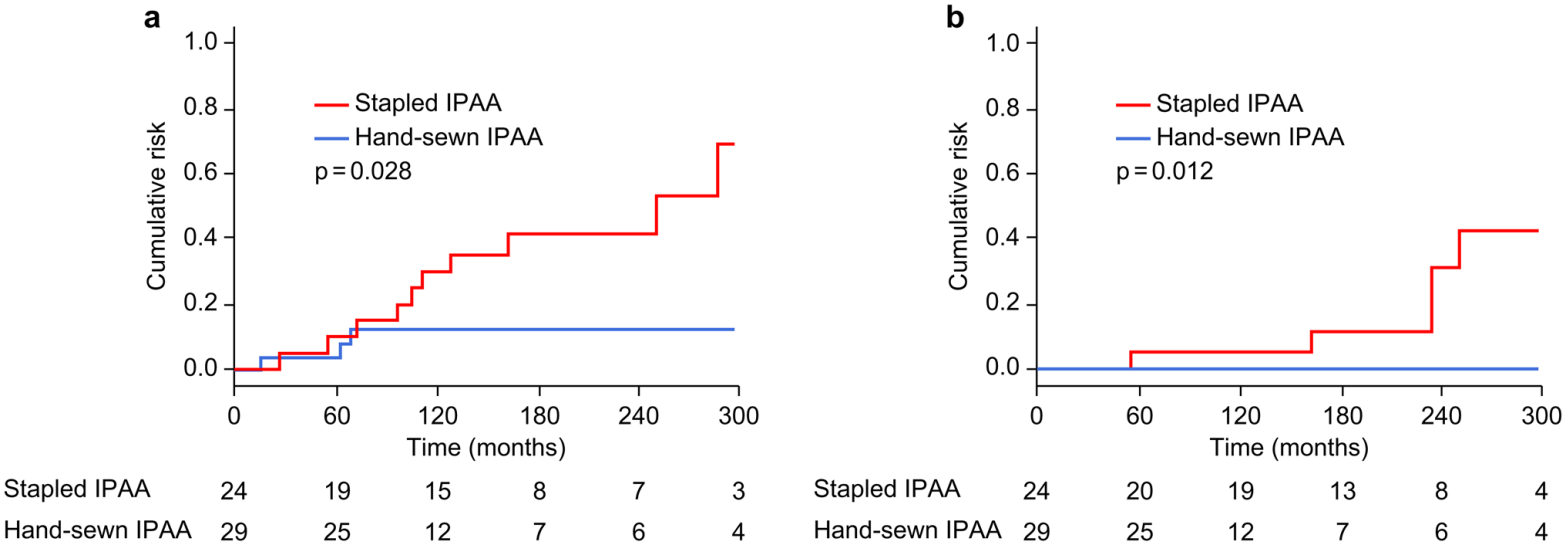
Cumulative risk of adenoma formation and metachronous carcinogenesis in the remnant rectum or anal transitional zone. **a** Comparison of adenoma formation. **b** Comparison of metachronous carcinogenesis. IPAA, total proctocolectomy with ileal pouch–anal anastomosis

The cumulative risk of adenoma incidence in the remnant rectum or ATZ is showed in Fig. 3a. This value was significantly higher in stapled than in hand-sewn IPAA (29.8% in stapled IPAA vs. 12.3% in hand-sewn IPAA at 120 months; 63.9% vs. 12.3% at 240 months, *p* = 0.028). The cumulative risk of metachronous remnant rectal cancer in the remnant rectum or ATZ is shown in Fig. 3b. After 20 years, this value was dramatically higher in stapled than in hand-sewn IPAA (5% in stapled IPAA vs. 0% in hand-sewn IPAA at 120 months; 31.0% vs. 0% at 240 months, *p* = 0.012).

### Patient survival

**Fig. 4:**
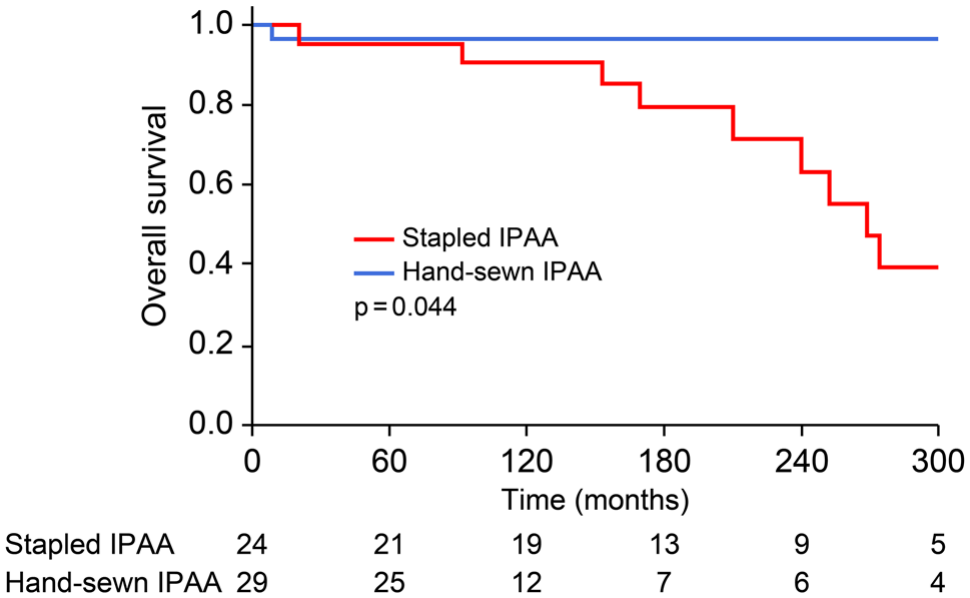
Comparison of overall survival IPAA, total proctocolectomy with ileal pouch–anal anastomosis

The Kaplan–Meier curves for OS are shown in Fig. 4. The OS in stapled IPAA was worse than that of hand-sewn IPAA (90.7% in stapled IPAA vs. 96.6% in hand-sewn IPAA at 120 months; 63.7% vs. 96.6% at 240 months, *p* = 0.044). Sensitivity analysis, excluding patients with interrupted follow-up, showed that the OS in stapled IPAA was worse than that in hand-sewn IPAA (*p* = 0.042, Supplementary Fig. 1).

The prognostic factors for FAP patients who underwent IPAA are presented in Table 3. In the univariable analysis, preoperative advanced colorectal cancer and the surgical procedure (stapled IPAA) were associated with worse OS (*p* = 0.019 and *p* = 0.026, respectively). In the multivariable analysis, preoperative advanced colorectal cancer (hazard ratio [HR], 4.738; 95% confidence interval [CI], 1.308–17.175; *p* = 0.019) and surgical procedure (stapled IPAA) (HR, 6.596; 95% CI, 1.216–122.288; *p* = 0.026) were independent predictors of poorer OS.

**Table 3:** Independent factors of clinical and surgical features affecting overall survival

	**Univariate analysis**			**Multivariate analysis**		
	**HR**	**95% CI**	***P* value**	**HR**	**95% CI**	***P* value**
Age at surgery (> 33 vs ≤ 33)	0.642	0.163–2.264	0.491			
Sex, male	1.134	0.313–4.108	0.843			
Family history	0.423	0.009–1.525	0.193			
Phenotype, profuse	0.525	0.113–1.900	0.335			
Preoperative early CRC	2.002	0.423–7.433	0.348			
Preoperative advanced colorectal cancer	4.637	1.285–16.733	0.021	4.738	1.308–17.175	0.019
Surgical procedure (stapled IPAA vs. hand-sewn IPAA)	6.509	1.196–120.95	0.027	6.596	1.216–122.388	0.026

*HR* hazard ratio, *CI* confidence interval, *FAP, CRC* colorectal cancer, *IPAA* ileal pouch–anal anastomosis

## Discussion

In this multicenter retrospective study, we analyzed the long-term outcomes of FAP patients who underwent stapled or hand-sewn IPAA. Twenty years after surgery, the OS of patients with stapled IPAA was dramatically worse than that of patients with hand-sewn IPAA. In the multivariable analysis, stapled IPAA was also associated with worse OS. To the best of our knowledge, this is the first study to show the effect of different anastomotic techniques in IPAA on the long-term outcomes of FAP patients. The findings of this study will have a significant impact on the choice of surgical procedure for FAP patients.

In many previous studies, most comparisons between stapled and hand-sewn IPAA have focused on postoperative complications or functional outcomes [21–23]. Regarding short-term prognosis, a previous national study in Japan has reported that the 5-year OS is similar between stapled and hand-sewn IPAA [14]. In the present study, the 5-year OS rates were similar between the two groups. The long-term postoperative outcomes of FAP patients have been reported, mainly in single-center, retrospective studies [24–26]. However, no study has directly compared stapled and hand-sewn IPAA regarding long-term prognosis, especially at 10 years or later postoperatively. In the present study, at 10 years or later postoperatively, the prognosis gradually worsened with stapled IPAA, with a dramatic difference in 20-year OS. We think that this result is due to the disease features of FAP, characterized by the transition from adenoma to carcinoma.

The presence of the remnant rectum and ATZ mucosa clearly differentiates stapled from hand-sewn IPAA [27]. Previous studies have reported the occurrence of adenomas in the remnant rectal mucosa of FAP patients, with cases of stapled IPAA coming up significantly more frequently than cases of hand-sewn IPAA [28–30]. The cumulative risk of adenoma in the remnant rectal mucosa has also been reported to be higher with stapled than with hand-sewn IPAA [15]. Similar results were observed in the present study, with a dramatic increase in the occurrence of adenomas in the remnant rectum and ATZ, particularly after 10 years or more postoperatively. Surprisingly, the cumulative incidence of metachronous remnant rectal cancer increased dramatically after about 20 years postoperatively. Metachronous remnant rectal carcinoma developing in the remnant rectum of FAP patients has a poor prognosis [31]. We believe that the occurrence of metachronous remnant rectal cancer leads to a poor prognosis for stapled IPAA. This trend is not observed in ulcerative colitis patients who undergo the same stapled or hand-sewn IPAA [32, 33]. Therefore, we believe that stapled IPAA is associated with a poor prognosis owing to the disease-specificity of FAP.

The occurrence rate of metachronous carcinogenesis in the remnant rectum or ATZ in this study tended to be higher than in previous studies [8, 29]. This difference would be influenced by the frequency with which pouchoscopies were performed. A recent report from a specialized center in Japan indicated that no evidence of metachronous carcinogenesis was found in the remnant rectum or ATZ over a median surveillance period of 11.5 years in patients who underwent annual pouchoscopies [29]. Similar findings have been reported by specialized facilities abroad [34]. However, some patients temporarily dropped out or refused to undergo the pouchoscopy despite the recommendation for annual pouchoscopies in this study. The observed extending the interval between surveillance could lead to a reduction in the number of adenoma treatments, potentially compromising the effectiveness of carcinogenesis prevention. Additionally, the inclusion of patients who dropped out of surveillance in this study may have contributed to the worse outcomes. Therefore, our findings emphasize the importance of continued postoperative surveillance.

The hand-sewn IPAA approach has some problems. Particularly, it should be noted that the risk of adenomas is not completely eliminated, even after hand-sewn IPAA with mucosectomy. Some studies have shown that mucosal islets of rectal mucosa may persist even after mucosectomy and hand-sewn anastomosis, while also estimating a 10–22.6% risk of adenoma development following hand-sewn IPAA [35, 36]. In the present study, the cumulative incidence of adenoma 10 years after hand-sewn IPAA was 12.3%. Fortunately, adenomas arising from the mucosal islands did not develop into carcinomas; however, with further surveillance, the possibility of carcinogenesis may arise. Future studies are needed to investigate the results of lifetime surveillance for FAP patients.

This study has a few limitations. First, this is a retrospective study with a small sample size. Our participants represent a clinic-based population from a single geographic region; therefore, this limits the generalizability of our experience and findings. Additionally, the retrospective design and small sample size of this study increase the risk of type 1 statistical errors and bias, necessitating cautious interpretation of the findings. Second, data on the detailed procedure of hand-sewn or stapled IPAA and the reason for choice of surgical procedure were not available. In a recent advanced study, long-term surveillance outcomes limited to patients with less than 20 rectal polyps who underwent total colectomy with ileorectal anastomosis showed that only 2% of patients developed metachronous remnant rectal cancer [37]. Therefore, there is a possibility of bias in the selection of the surgical procedure in this study. Third, this study did not evaluate postoperative quality of life. Hand-sewn IPAA is known to decrease postoperative quality of life by leading to issues such as fecal incontinence [38]. The findings of this study should be used to guide clinical practice with great caution. Finally, this study period spanned a long time, during which changes in the treatment of patients, including advancements in surgical techniques, endoscopic technology, and improvements in postoperative surveillance management, may have occurred.

## Conclusion

In conclusion, long-term surveillance revealed that stapled IPAA negatively affected the OS of FAP patients. Therefore, we strongly recommend hand-sewn IPAA over stapled IPAA for a better prognosis in FAP patients.

## Supplementary Information

Below is the link to the electronic supplementary material.

Supplementary file1 (DOCX 108 KB)

## Data Availability

The datasets used and/or analysed during the current study are available from the corresponding author on reasonable request.
