# Supplementary material for: Long-term prognosis after stapled and hand-sewn ileal pouch–anal anastomoses for familial adenomatous polyposis: a multicenter retrospective study
Source: Int J Colorectal Dis. 2024 Mar 2;39(1):32. doi: 10.1007/s00384-024-04608-9 (PMC10908628; doi:10.1007/s00384-024-04608-9)
Supplement: Supplementary file 1 — Supplementary file1 (DOCX 108 KB) [file 384_2024_4608_MOESM1_ESM.docx]

**Comparison of Overall Survival after Stapled and Hand-sewn Ileal Pouch–Anal Anastomoses for Familial Adenomatous Polyposis: A Multicenter Retrospective Study**

Kyota Tatsuta^1^, Mayu Sakata^1^*, Moriya Iwaizumi^2^, Kazuya Okamoto^3^, Shigeto Yoshii^4^, Masashi Mori^5^, Yutaro Asaba^6^, Takashi Harada^7^, Mikihiro Shimizu^8^, Kiyotaka Kurachi^1^, and Hiroya Takeuchi^1^

^1^Department of Surgery, Hamamatsu University School of Medicine, 1-20-1, Handayama, Higashi-ku, Hamamatsu, Shizuoka 431-3192, Japan

^2^Department of Laboratory Medicine, Hamamatsu University School of Medicine, 1-20-1 Handayama, Higashi-ku, Hamamatsu Shizuoka 431-3192, Japan

^3^Department of Surgery, Fujieda Municipal General Hospital, 4-1-11 Surugadai, Fujieda, Shizuoka 426-8677, Japan

^4^Department of Gastroenterology, Fujieda Municipal General Hospital, 4-1-11 Surugadai, Fujieda, Shizuoka 426-8677, Japan

^5^Department of Gastroenterology, Fujinomiya City General Hospital, 3-1 Nishiki-cho, Fujinomiya Shizuoka 418-0076, Japan

^6^Department of Surgery, JA Shizuoka Kohseiren Enshu Hospital, 1-1-1 Chuou, Naka-ku, Hamamatsu 430-0929, Japan

^7^Department of Surgery, Hamamatsu Medical Center, 328, Tomitsuka, Naka-ku, Hamamatsu, Shizuoka 432-8580, Japan

^8^ Center for Clinical Research, Hamamatsu University Hospital, 1-20-1 Handayama, Higashi-ku, Hamamatsu, Shizuoka 431-3192, Japan

***Corresponding Author** **Email**: mayu-s@hama-med.ac.jp

**SUPPLEMENTARY TABLES AND FIGURES**

| **Surgical procedure** | ***APC* variant** | **Classifications** | **Criteria codes** |
| --- | --- | --- | --- |
| Stapled IPAA | NM_000038.6(APC):c.3927_3931del (p.Glu1309fs) | pathogenic | PVS1, PS4_very strong, PM6_very strong |
| Stapled IPAA | NM_000038.6(APC):c.3329C>G (p.Ser1110Ter) | likely pathogenic | PVS1, PS4moderate |
| Stapled IPAA | NM_000038.6(APC):c.3747C>A (p.Cys1249Ter) | pathogenic | PVS1,PM2 supportive, PS4 moderate |
| Stapled IPAA | NM_000038.6(APC):c.832C>T (p.Gln278Ter) | pathogenic | PVS1, PS2 |
| Hand-sewn IPAA | NM_000038.6(APC):c.1495C>T (p.Arg499Ter) | likely pathogenic | PVS1, PS4 moderate |
| Hand-sewn IPAA | NM_000038.6(APC):c.3183_3187del (p.Lys1061_Gln1062insTer) | likely pathogenic | PVS1, PS4 moderate |
| Hand-sewn IPAA | NM_000038.6(APC):c.834+2T>C | pathogenic | PVS1, PS4, PS2 |
| Hand-sewn IPAA | NM_000038.6(APC):c.637C>T (p.Arg213Ter) | pathogenic | PVS1,PS2, PS4 moderate |
| Hand-sewn IPAA | NM_000038.6(APC):dup(ex2-3) | pathogenic | PVS1, PS4 |
| Hand-sewn IPAA | NM_000038.6(APC) c.(?_-380)＿(*2113-?) | pathogenic | PVS1, PS2 |
| Hand-sewn IPAA | NM_000038.6(APC):c.2547_2548del (p.Asp849fs) | pathogenic | PVS1, PS4 moderate |

**Supplementary Table. 1** The characteristics of variants in genetically diagnosed cases

IPAA, total proctocolectomy with ileal pouch-anal anastomosis

APC, adenomatous polyposis coli

PVS, pathogenic very strong

PS, pathogenic strong

PM, pathogenic moderate

**
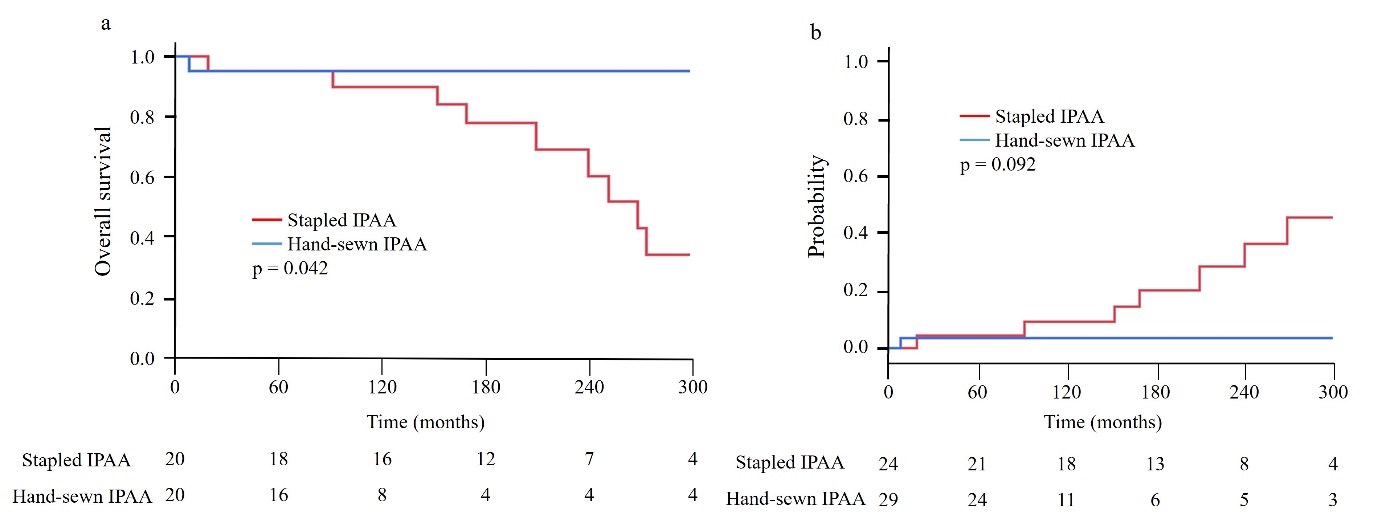
**

**Supplementary Fig. 1** Additional analysis of long-term prognosis

(a) Sensitivity analysis for eligible patients after excluding those with interrupted follow-up. (b) Gray test accounting for deaths unrelated to FAP-related malignancies or surgery as a competing risk. IPAA, total proctocolectomy with ileal pouch-anal anastomosis
